# What Makes a Photobattery Light-Rechargeable?

**DOI:** 10.1021/acsenergylett.4c01350

**Published:** 2024-07-23

**Authors:** Arvind Pujari, Byung-Man Kim, Hooman Abbasi, Myeong-Hee Lee, Neil C. Greenham, Michael De Volder

**Affiliations:** † Cavendish Laboratory, Department of Physics, University of Cambridge, Cambridge CB3 0HE, United Kingdom; ‡ Institute for Manufacturing, Department of Engineering, University of Cambridge, Cambridge CB3 0FE, United Kingdom; § School of Energy and Chemical Engineering, Ulsan National Institute of Science & Technology, Ulsan 44919, South Korea

## Abstract

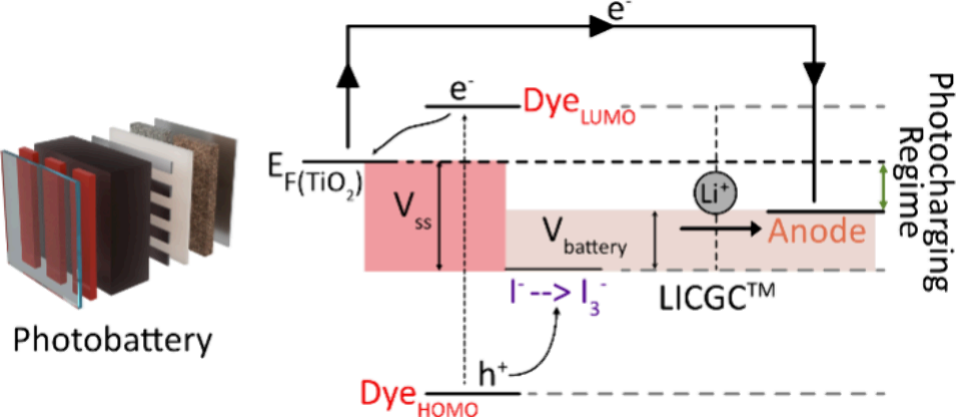

The demand for autonomous off-grid devices has led to the development of “photobatteries”, which integrate light-energy harvesting and electrochemical energy storage in the same architecture. Despite several photobattery chemistries and designs being reported recently, there have been few insights into the physical conditions necessary for charge transfer between the photoelectrode and counter electrode. Here, we use a three-electrode photobattery with a dye-sensitized TiO_2_ photoelectrode, triiodide (I^–^/I_3_^–^) catholyte, and anodes with varying intercalation potentials to confirm that photocharging is only feasible when the conduction band quasi-Fermi level (E_Fc_) is positioned above the anode intercalation/plating potential. We also show that parasitic reactions after the battery is fully charged can be accelerated if the voltage of the battery and solar cell are not matched. The integration of multiple anodes in the same photobattery ensures well-controlled measurement conditions, allowing us to demonstrate the physical conditions necessary for charge transfer in photobatteries, which has been a topic of controversy in the field.

The rise of the Internet of Things (IoT), smart cities and industry 4.0 has led to the development of networks of off-grid smart sensors.^1^ Solar energy (through indoor or outdoor light) has emerged as one of the principal sources of energy to power these off-grid devices. However, solar cells must be coupled with energy storage devices such as batteries to allow for a continuous power supply. To reduce the footprint of these devices, efforts are underway to integrate solar cells and batteries within the same device architecture, which are commonly referred to as ‘light-rechargeable photobatteries’.^2^

Since the 1970s, various three-electrode photobatteries have been developed, where a solar cell and a battery share an electrode. One of the first reported systems involved a cadmium selenide (CdSe) photoabsorber, sulfur redox couple (S/S^2–^) and a silver anode which underwent an alloying reaction with sulfur to form Ag_2_S.^3^ Other efforts include a system consisting of a dye-sensitized photoelectrode, iodide catholyte and WO_3_ anode,^4^ a system utilizing perovskite solar cells that incorporate zinc-ion battery cathode layers as their hole transport material (HTM)^5^ and a system based on dye-sensitized solar cells (DSSC) with a copper complex catholyte and LiMn_2_O_4_ (LMO) anode designed for indoor light charging of batteries.^6^ These devices require the connection of the photoelectrode and anode (storage electrode) while charging, and a separate electrode is used for discharge.

After initial efforts in the 1980s^7^ and 1990s,^8^ there have been efforts in recent years to combine the photoelectrode and discharge electrode in devices known as two-electrode photobatteries. Here, a semiconductor material can either serve as the battery cathode (bifunctional cathodes), or is mixed with a semiconductor material, thus combining the functions of light-harvesting and energy storage within a single electrode.^2^ Examples include a dye-sensitized LiFePO_4_ cathode coupled with a Li metal anode which was photocharged to 3.5 V upon illumination through side-reactions involving the electrolyte,^9^ a perovskite photocathode paired with a lithium metal anode^10^ and a V_2_O_5_–P3HT cathode cycled against a zinc metal anode.^11^

Irrespective of the system, a crucial requirement for photo charging is favorable band alignment between the light-absorber and the anode in order to enable charge transfer between the electrodes.^12^ Nevertheless, the exact energetic requirements for photocharging remain a matter of debate in the field, with several papers presenting photocharging in systems where the photoactive element appears to provide insufficient potential to drive the primary charging reaction claimed.^9,10,13−26^ Here, we use a 3-electrode system consisting of a dye-sensitized TiO_2_ photocathode, triiodide catholyte (I^–^/I_3_^–^) and Li_1+x_Mn_2_O_4_/LiMn_2_O_4_ (LMO) or Li_4_Ti_5_O_12_/Li_7_Ti_5_O_12_ (LTO) anodes to study charge transfer based on energy level alignment in light-rechargeable photobatteries. A schematic of the system is provided in Figure 1 showing that the device is composed of a dye-sensitized solar cell (DSSC) component and a battery component. A lithium-ion-conducting glass-ceramic (LICGC, OHARA GmbH) serves as the link between these two, allowing for lithium-ion conduction between them during charge and discharge of the battery.

**Figure 1:**
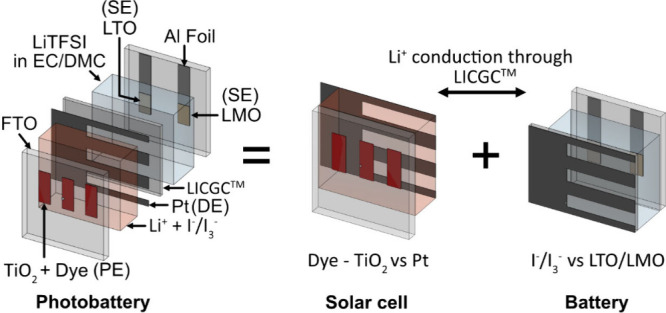
A schematic of the three-electrode photobattery used in this study. The device consists of a solar cell component (dye sensitized TiO_2_, a I^–^/I_3_^–^ mediator and a Pt counter electrode) and a battery component (I^–^/I_3_^–^ catholyte and LTO/LMO anodes with a LiTFSI based electrolyte). The solar cell and the battery are connected through a Li-ion conductive ceramic separator, enabling the transfer of photogenerated electrons and lithium ions between the solar cell and the battery during charge and discharge cycles.

In a classic battery operation mode, the triiodide mediator acts as a catholyte, changing its oxidation state from I^–^ to I_3_^–^ during charging. To maintain charge neutrality, Li ions, which are codispersed in the catholyte, are transferred through the LICGC separator and are stored in the anode. As discussed above, two anodes with different operating potentials are integrated in the same device to test the effect of the counter electrode potential under the exact same conditions.

In a classic DSSC operation mode, a dye layer adsorbed on the surface of TiO_2_ absorbs light which excites an electron from its HOMO (highest occupied molecular orbital) to its LUMO (lowest unoccupied molecular orbital). The excited electron is then injected from the LUMO to the conduction band of TiO_2_, while the hole in the HOMO oxidizes the redox mediator. The open-circuit voltage of the solar cell is the difference between the conduction-band quasi-Fermi level for electrons in TiO_2_ (E_Fc_) and the redox mediator potential. The detailed operation of the combined battery - DSSC system is discussed later.

We demonstrate the attempted photocharging of two anode materials with differing lithiation potentials (LMO and LTO) and show that photocharging can only proceed when the E_Fc_ of the photoabsorber lies at a higher energy than the anode intercalation reaction. We also vary the E_Fc_ of the photoabsorber by changing the light intensity and show that as long as E_Fc_ is higher than the anode intercalation potential, charge transfer will proceed until the battery is fully charged. We show that parasitic reactions are possible once the battery is fully charged, and it is necessary to match the voltage of the solar cell and battery to prevent this.

Thus, these results demonstrate the physical requirement for photocharging phenomena in photobatteries. Although we use a 3-electrode system with a dye-sensitized photoelectrode (PE) to precisely control the energy level alignment between the PE and anode storage electrode (SE), the conclusions drawn from this study are also applicable to two-electrode photobatteries as the physics of charge transfer between the two electrodes remains the same.

The structure of the device used to study energy level alignment is shown in Figure 2(a) and consists of a PE, discharge electrode (DE) and SE. The solar cell component of the device comprises the PE and DE whereas the terminals of the battery component are the DE and SE. The PE consists of a mesoporous TiO_2_ layer cosensitized with three organic dyes (WS72, MS5, and XY1b (Dyenamo AB)). The structure of the dyes is provided in Figure S1(a-c). We mainly used a modified version of the Y123 dye (WS72), which was designed to minimize energy loss during light energy conversion.^27^ XY1b^28^ and MS5^29^ dyes were introduced as cosensitizers to extend spectral absorption range and to minimize the interfacial charge recombination, respectively. The absorbance spectra of the dyes are shown in Figure 2(d). A redox mediator (I^–^/I_3_^–^, M_Red_/M_Ox_) (with 0.1 M LiI as a lithium-ion source) with a redox potential of (≈ 0.4 V vs SHE, Figure S2) is used both as the redox couple for the solar cell and catholyte of the battery.

**Figure 2:**
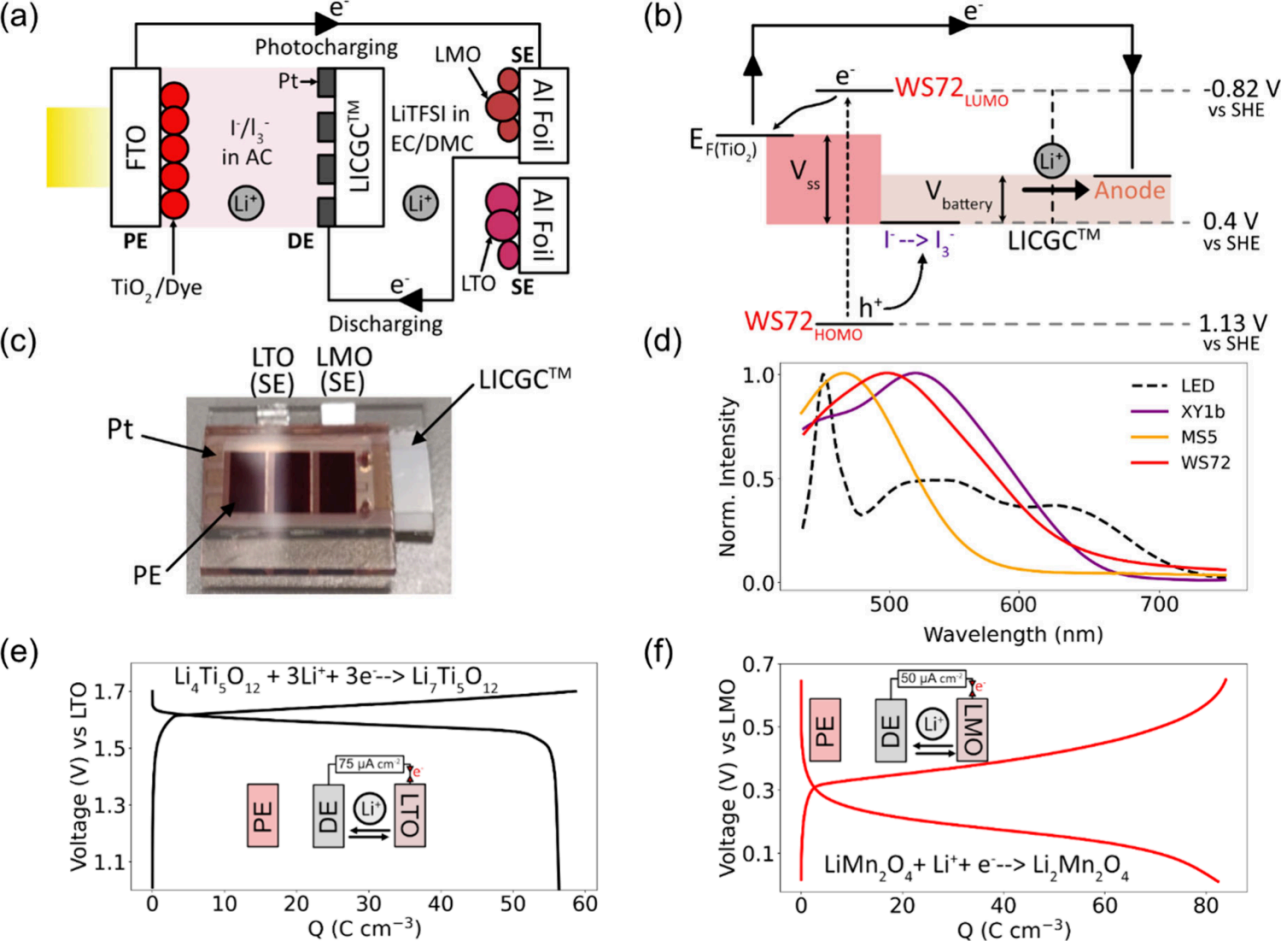
Schematic of the three-electrode photobattery used in this study, working mechanism and electrochemical performance. (a) The device is made up of dye sensitized TiO_2_ as the photoelectrode, patterned Pt sputtered on a lithium-conductive glass (LICGC) as the discharge electrode (DE) and LMO/LTO as the storage electrode (anode/SE). The solar cell electrolyte is a mixture of a I^–^/I_3_^–^ redox mediator and a Li^+^ ion source. The battery electrolyte is 1 M LiTFSI in EC/DMC (1:1). (b) To photocharge the battery, the PE and SE are electrically connected under illumination resulting in photogenerated electrons being transferred from the dye to the TiO_2_ layer and then, to the anode. Meanwhile, photogenerated holes oxidize the mediator resulting in Li^+^ diffusion through the LICGC separator to the battery where it combines with the electron to insert into the anode. During discharge, the SE and DE are connected, and the process is reversed. The energy levels in the schematic are not to scale. (c) An image of the device structure. (d) The absorption spectra of the three dyes and the spectrum of the white LED used for illumination. (e) Electrochemistry of the LTO vs I^–^/I_3_^–^ battery with a nominal charging voltage of 1.65 V. (f) Galvanostatic charge–discharge curves of the LMO vs I^–^/I_3_^–^ battery with a nominal charging voltage of 0.4 V.

The counter electrode of the solar cell is Pt sputtered in a patterned fashion (Figure S3) on a lithium-conductive glass (LICGC). The LICGC membrane allows for the separation of the mediator from the storage electrode (SE, anode) of the battery–if this were not the case the oxidized mediator would spontaneously oxidize the SE, short-circuiting the battery. The space between LICGC and the SE is filled with a classic battery electrolyte: 1 M LiTFSI in ethylene carbonate (EC): dimethyl carbonate (DMC) (1:1). We consider two SEs (anodes). The first is LMO (intercalation potential of 3 V vs Li^+^/Li or 0 V vs SHE) graphitized by ball milling–the surface treatment stabilizes the structure to enable lithium insertion into LMO at 3 V vs Li^+^.^30−32^ This is due to the formation of nanosized grains during the ball milling treatment. This results in less anisotropic strain being generated which minimizes cracking due to severe Jahn–Teller distortion in the crystals.^33^ Additionally, the graphite coating improves electrical connections between particles. The second SE is LTO (intercalation potential of 1.75 V vs Li^+^/Li or −1.25 V vs SHE). Our cell contains two separate SEs in the same device, as described earlier, which allows for a precise comparison of anodes with different intercalation potential.

The working mechanism of the device is shown in Figure 2(b). During photocharging, the PE and SE are electrically connected, and the PE is illuminated, resulting in electron–hole pairs being generated in the dye. The HOMO level of the dye is obtained from cyclic voltammetry (Figure S4, + 1.13 V vs SHE), with the LUMO calculated as E_HOMO_ + E_Band Gap_. Since the LUMO of the dye is located above (more negative vs SHE) the E_Fc_ of TiO_2_, electrons are injected from the dye into TiO_2_. The HOMO of the dye is located below the redox potential of the I^–^/I_3_^–^ mediator allowing the photogenerated holes to trigger the I^–^/I_3_^–^ transition.^34^ A Li^+^ ion from the battery electrolyte combines with the photogenerated electron from the PE to intercalate into the anode. For charge compensation, a Li^+^ ion is conducted from the electrolyte (which contains 0.1 M LiI) through the regions of LICGC separator that are not sputter coated with Pt to the battery component of the device. Thus, the photocharging reaction is as follows:
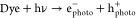

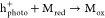

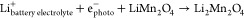

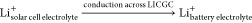
where *M*_*ox*_ and *M*_*Red*_ are the oxidized and reduced forms of the triiodide mediator (I^–^/I_3_^–^), respectively. During discharge, a constant current is applied between the SE and DE. Electrons from the SE flow to the DE where they reduce I_3_^–^ to I^–^ on the surface of the catalytic Pt layer. For charge compensation, a Li^+^ ion is conducted across the LICGC separator to complete the discharge process. Hence, our device uses a I^–^/I_3_^–^ redox couple as a catholyte which is paired against an LTO or LMO anode. The voltage generated by the solar cell is determined by the difference between the E_Fc_ of TiO_2_ and the I^–^/I_3_^–^ redox couple potential. In contrast, the battery potential is the difference between the intercalation potential of the SE and the Nernst potential of the I^–^/I_3_^–^ redox couple. Thus, the electrochemical reaction during discharge is
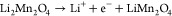

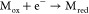

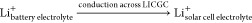
During electrochemical charging, the mechanism of the battery operation is the same as for photocharging, except that the mediator is oxidized by applying a potential between the Pt (on the DE) and the anode (on the SE).

The galvanostatic charge–discharge profiles of the I_3_^–^/I^–^ vs LTO battery and the I_3_^–^/I^–^ vs LMO battery are shown in Figure 2(e) and (f) respectively. The batteries are charged and discharged by applying a constant current between the DE and SE. Although a single catholyte (I_3_^–^/I^–^) is used, the presence of two separate anodes allows two different cell chemistries to be measured within the same device, as shown in Figure 2(c). During charging, the iodide catholyte is oxidized and lithium is inserted into the SE, with the reverse occurring during discharge. The nominal charging voltage of LTO as calculated from the charge–discharge curves is −1.65 V vs I_3_^–^/I^–^ (1.75 V vs Li^+^/Li) or −1.25 V vs SHE while that of LMO is −0.4 V vs I_3_^–^/I^–^ or 0 V vs SHE (3 V vs Li^+^/Li). As the conduction band edge (which is close to E_Fc_) of TiO_2_ is typically recorded as −0.5 V SHE^35^, these anodes enable us to investigate photocharging behavior depending on whether E_Fc_ is higher or lower than the intercalation potential of the anode.

For a DSSC, the relationship between the cell open-circuit voltage (*OCV*_*solar cell*_), conduction band quasi-Fermi level of TiO_2_ (*E*_*Fc*_) and redox potential of the mediator (*E*_*redox*_) is given by^36^
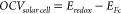


The J-V curve for the solar cell component of the photobattery was obtained under 1 sun by masking the solar cell to an active area of 0.04 cm^2^ as shown in Figure 3(a) as this provided sufficient current to charge the battery in a reasonable amount of time. All photocharging experiments at this light intensity were carried out using the same active area of the solar cell. The OCV obtained from the JV curve enabled us to construct the energy diagram as shown in Figure 3(b). The value E_Fc_ for TiO_2_ was calculated to be −0.34 V vs SHE from the J-V curve which was higher than the nominal voltage for Li^+^ intercalation into LMO (0 V vs SHE) but lower than that of LTO (−1.25 V vs SHE) as shown in Figure 3(b).

**Figure 3:**
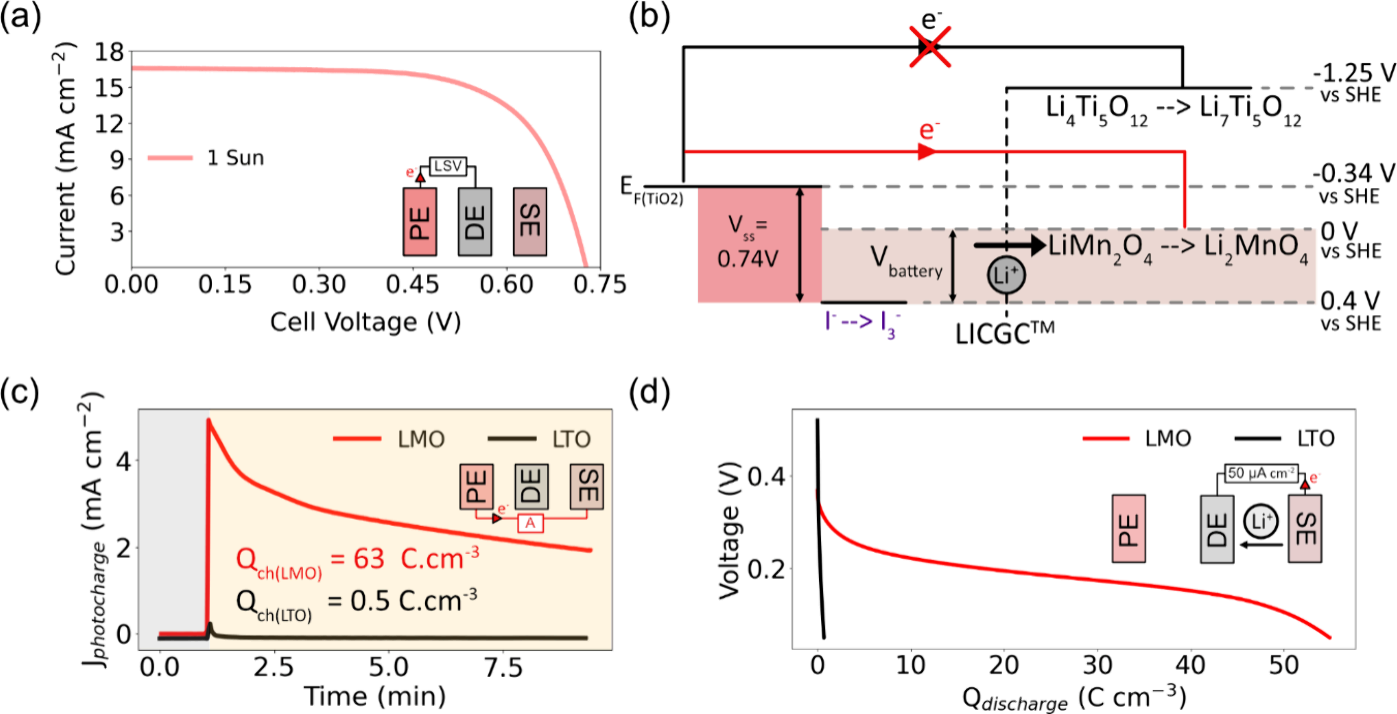
Photocharging LTO and LMO SEs. (a) The J-V curve of the solar cell component of the photobattery was measured by connecting the PE and DE. (b) The energy diagram of the photobattery shows that the intercalation potential of LTO is higher (more negative) than E_Fc_ while the converse is true for LMO, allowing us to test two energy regimes. The magnitudes of the energy levels are not to scale. (c) Photocharging was carried out by electrically connecting the PE and the two SEs through a potentiostat. The solar simulator was switched off in the gray shaded region and on in the yellow shaded region. The LMO SE exhibited an initial charging photocurrent of ∼5 mA cm^–2^ and a charged capacity of 63 C cm^–3^, whereas the LTO SE displayed an initial charging photocurrent of only ∼0.1 mA cm^–2^ and a charged capacity of 0.5 C cm^–3^. (d) Both SEs were discharged by applying a constant current between the SE and DE. Discharge capacities of 57 C cm^–3^ and 0.7 C cm^–3^ were obtained from the photocharged LMO and LTO SEs, respectively, indicating that only LMO had been effectively charged due to its favorable energy level alignment.

To photocharge the LMO anode, the PE and LMO SE were connected and then the PE was illuminated until a charging capacity of 63 C cm^–3^ was obtained as shown in Figure 3(c). This cutoff capacity was based on the capacity of the cell when charged to 0.4 V. A high current of ≈5 mA cm^–2^ was initially seen which reduced to ≈2 mA cm^–2^ by the end of photocharging. In contrast, when photocharging cells with LTO anodes, a small initial photocurrent of 0.1 mA cm^–2^ was observed which reduced to less than 7 μA cm^–2^ within 10 s. The current remained at this magnitude throughout the photocharging period, which was the same duration as that used for photocharging LMO, resulting in a charged capacity of 0.5 C cm^–3^. This indicates that negligible charge was transferred to LTO, and no photocharging takes place. The small photocharging current briefly seen is likely to correspond to a small amount of capacitive charging between 0 and 0.7 V for LTO.

When the two SEs were discharged using a constant current of 50 μA cm^–2^ after photocharging, as shown in Figure 3(d), a capacity of 57 C cm^–3^ was obtained for the LMO SE which is ≈90% of the photocharged capacity. However, for the LTO anode, a discharge capacity of 0.7 C cm^–3^ was obtained which is almost negligible. Both the photocharging and discharge capacities were 2 orders of magnitude lower than that of LMO, indicating that LTO was not photocharged in this process. This is because its E_Fc_ is below (less negative vs SHE) the intercalation potential of LTO.

To confirm that the photogenerated capacity originated from the intercalation of lithium-ions into LMO, we performed cyclic voltammetry to determine the redox potentials of LMO and I_3_^–^/I^–^(Figure S5). This revealed that the potential of this reaction is about +0.23 V vs SHE, which is similar to the discharge potential seen in Figure 3(d) and Figure S6. This is a strong indication that the photocharging reactions taking place in the batteries are indeed the same as the ones we tested during galvanostatic charge and discharge.

Additionally, we carried out photocharging for 5 consecutive cycles at different light intensities (1 sun, 2000 lx, 1000 lx, 500 and 200 lx) with Coulombic efficiencies between 94% - 97% seen for all cycles, as shown in Figure S6. Thus, photocharging occurs at the right potential for intercalation into LMO, with a high Coulombic efficiency, indicating that most of the photogenerated charge is used for reversible intercalation into LMO and not a side reaction, proving our photocharging mechanism.

We also demonstrate photocharging under both 1 sun and indoor light intensities. Given the extremely low intensities under indoor light (200 lx ≈0.0006 sun), thermal effects are negligible under these conditions, indicating that the photocurrent seen is due to electron–hole pair generation under illumination and not heating of the photobattery.

Thus, we demonstrate the physical requirements for photocharging of a battery to be possible, namely, E_Fc_ should be higher than the anode intercalation potential. However, several publications have reported the photocharging of cathodes where the conduction band potential (which can be approximated to the potential of E_Fc_), is lower than the anode plating/intercalation reaction, as summarized in Table S1. In contrast to these reports, here we demonstrate that charge transfer between the photoelectrode and anode is not possible under these conditions. Hence, it is imperative that alternate mechanisms are put forward to address this apparent “photocharging” phenomenon. For example, Pan et al.^24^ proposed that trace amounts of atmospheric oxygen may be leaking through the window of the photobattery and participating LiO_2_ and Li_2_O_2_ formation on the cathode, resulting in a change in cell OCV, whereas Mathieson et al.^37^ proposed the buildup of a capacitive double layer due to photogenerated holes on the cathode.

We next show that if the voltage of the solar cell and battery are not matched, overcharging can occur. To do this, we control the E_Fc_ of TiO_2_ by varying the light intensity used.^38^ A white LED was used to obtain precise control over the light intensity and the entire area of the solar cell (0.96 cm^2^) was used during photocharging. The value of E_Fc_ was calculated from the JV curve in Figure 4(a) to be −0.23 V vs SHE under 200 lx illumination and −0.30 V vs SHE under 1000 lx illumination (this is equivalent to about 0.003 sun). This is shown in the energy diagram depicted in Figure 4(b).

**Figure 4:**
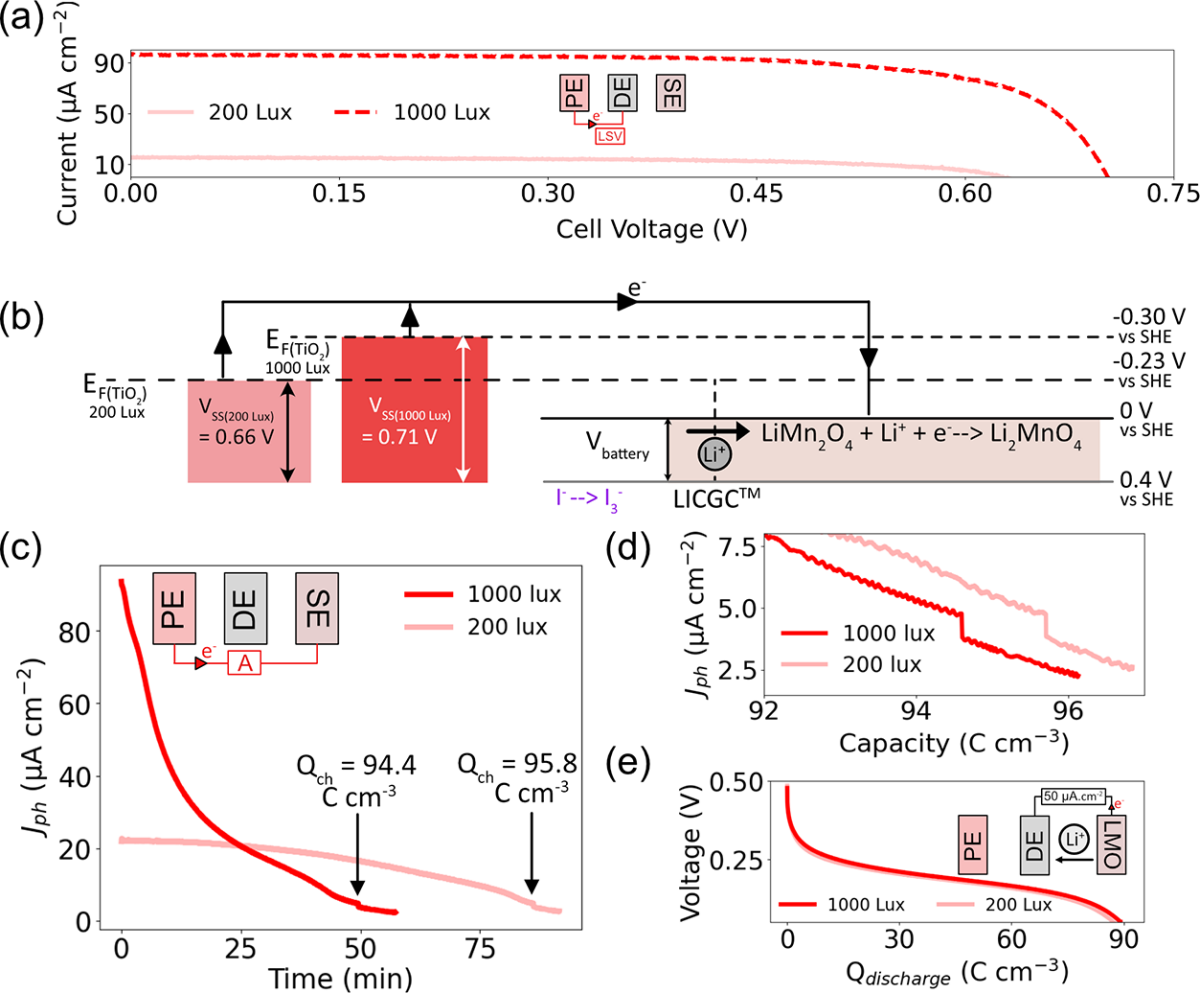
(a) J-V curves (obtained through linear-sweep voltammetry (LSV)) of the solar cell components of the photobattery under 200 and 1000 lx illumination by a white LED. (b) The energy diagram of the device under 1000 and 200 lx. The magnitudes of the energy levels depicted are not to scale. (c) The PE and LMO SE were short circuited and illuminated, with the photocharging current recorded under 1000 and 200 lx light intensities for an extended period. Both curves showed a sudden dip in photocharging current at about 95 C cm^–3^ which was indicative of the maximum capacity of the cell being reached. (d) A zoomed in plot of photocharging current vs charged capacity, highlighting that the drop in photocharging current occurred under both light intensities. (e) Constant current discharge curves after photocharging, with a capacity of about 89 C cm^–3^ recorded in both cases.

We charged the batteries for an extended period under both light intensities, with the results shown in Figure 4(c). The measured photocurrent steadily decreased under both conditions, with a longer charging time observed under 200 lx due to lower current generation from the solar cell. A sharp decrease in current was seen in both cases: after about 50 min under 1000 lx illumination and 90 min under 200 lx illumination. The current vs capacity plots in Figure 4(d) show that the decrease in current occurs after a charged capacity of about 95 C cm^–3^ in both cases. A similar decrease in photocharging current can be seen under 1 sun illumination as shown in Figure S7(a). This decrease in current likely corresponds to the maximum charge capacity of the anode being reached.

Upon discharge, capacities of 88.5 C cm^–3^ and 89.0 C cm^–3^ were obtained after 200 and 1000 lx charging respectively, as shown in Figure 4(e), while a capacity of 90.2 C cm^–3^ was obtained after long-term photocharging under 1 sun (Figure S7(b)). The discharge capacity and maximum photocharged capacity (corresponding to the point of sharp current decrease) were similar to the maximum capacity of the battery obtained during galvanostatic charge–discharge experiments, indicating that if the Fermi level is high enough, the maximum capacity of the cell can be reached. The slight increase in maximum capacity when compared to the results in Figure 2 is due to activation of the LMO anode.^30^

However, under both 200 and 1000 lx illumination, it was possible to overcharge the cell past its point of maximum capacity. It should be noted that the Coulombic efficiency in the above tests was around 94%, which indicates that side reactions are occurring throughout the charging process. However, after the completion of charging there remained a parasitic current of ≈3 μA cm^–2^ (equivalent to ≈0.03 C) under both 200 and 1000 lx illumination which might drive side reactions. This corresponds to extra capacities of 1.7 C cm^–3^ and 1.3 C cm^–3^ (after 5 min of overcharging) under 200 and 1000 lx, respectively.

This effect was more pronounced at high light intensities. After the completion of photocharging under 1 sun (Figure S7(a)) an initial parasitic current of 33 μA cm^–2^ (equivalent to ≈0.3 C) was observed, which decreased to 5.5 μA cm^–2^ after 20 min. This corresponds to an extra capacity of 8.5 C cm^–3^ after 5 min of overcharging (about 9% of the cell capacity) indicating that side reactions are more visible when operating at stronger light intensities due to the solar cell generating greater current. Thus, identifying the reaction potentials of possible side reactions is a key consideration to maximize the performance of photoelectrochemical energy storage systems.

Finally, we discuss some of the considerations for voltage (V) – current (I) matching in photobatteries. Figure 5(a) shows the voltage vs time plots for the I^–^/I_3_^–^ vs LMO battery used in this study. Three different times are marked on the plot: *t*_1_ (corresponding to 2% state of charge (SoC)), *t*_*2*_ (40% SoC) and *t*_*3*_ (100% SoC). At any given state of charge, a battery will have a current–voltage curve that we will model as an ideal voltage source (the open-circuit voltage) in series with an impedance (the internal resistance). When the battery and solar cell are connected for photocharging, their I–V curves must intersect, i.e. they should operate at the same voltage and current. To construct the I–V curves of the battery we recorded the photocharging currents at different SoCs and obtained the corresponding battery voltage from the voltage vs time plot in Figure 5(a). This plot was obtained at a constant current charging rate of 0.4 C which is ≈3.1 times (on average) slower than photocharging, thus charging the cell close to its equilibrium potential.

**Figure 5:**
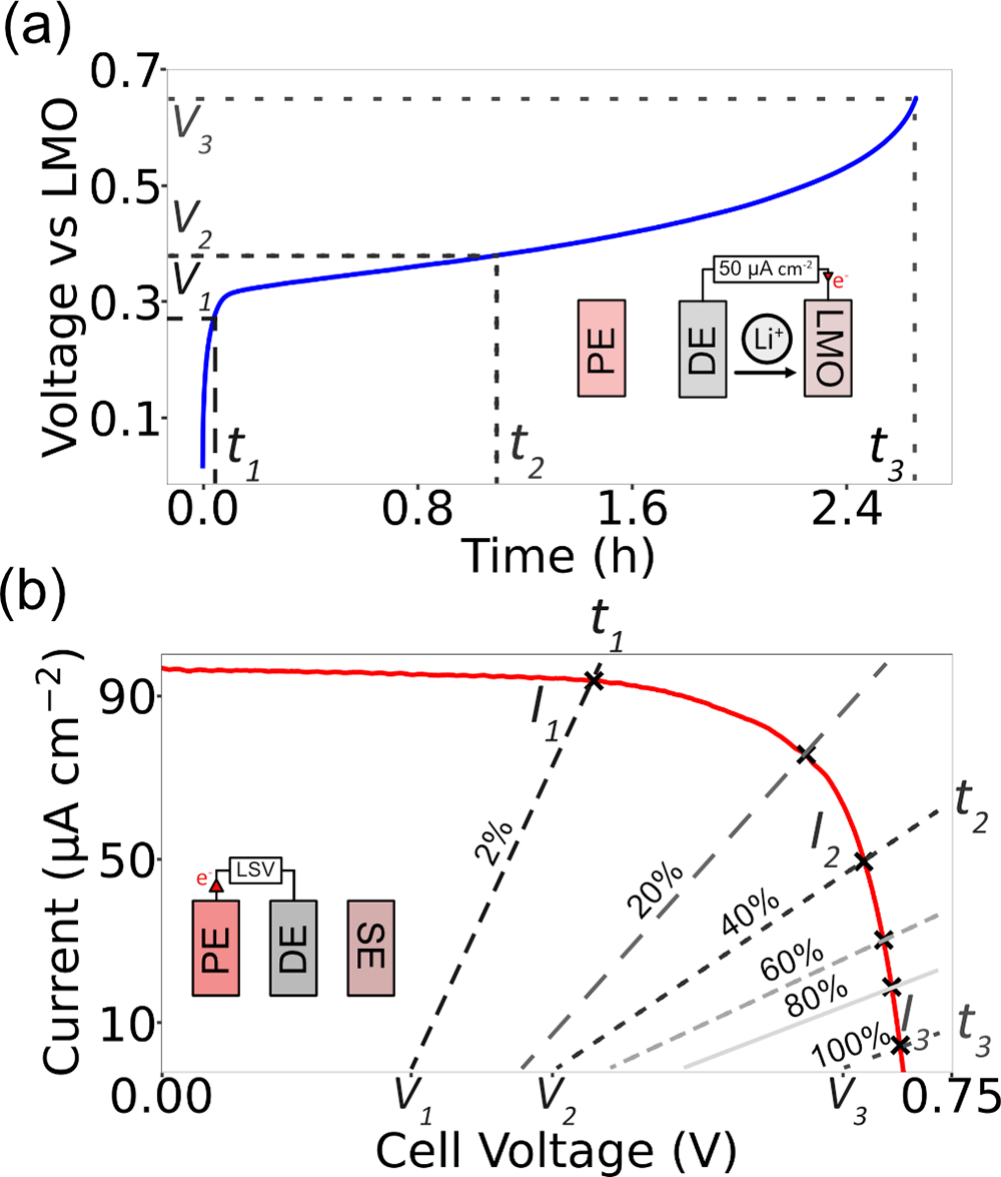
Considerations for voltage matching in photobatteries. (a) Voltage vs time plot (at a constant-current charging rate of 0.4 C) for the I^–^/I_3_^–^ vs LMO battery used in this study. (b) Current vs voltage plot for the solar cell at 1000 lx. At any given SoC, we model the battery as an ideal voltage source with an impedance, as indicated by it is I–V curves (dashed lines). When photocharging occurs, the solar cell and battery operate at the same current and voltage (their I–V curves intersect), with the current determined by the output (photocharging) current of the solar cell at that voltage, minus some losses. As the battery charges, its voltage increases, and the I–V curve of the battery shifts to the right, reducing the output current from the solar cell. The resistance of the battery also changes (shown by the changed slope of the battery I–V curve), reducing the photocharging current.

It should be noted that the I–V curve of the solar cell is also likely to vary with state-of-charge (SoC) due to changes in lithium-ion and mediator concentration, we only consider changes in the battery resistance in this analysis. Therefore, a decrease in the value of the output solar cell current as a function of SoC is also a contributing factor to the decrease in photocurrent, a sketch of which is shown in Figure S8.

We then superimpose the I–V curves of the battery at *t*_1,_*t*_2_ and *t*_3_ on the I–V curve of the solar cell as shown by the dashed lines in Figure 5(b). The slope of the I–V curve represents the internal resistance of the battery. The intersection of the I–V curve of the battery with that of the solar cell indicates the current that is being provided by the solar cell to the battery at that instant, as shown by the currents *I*_1,_*I*_2_, and *I*_3_ at times *t*_1,_*t*_2_ and *t*_3_ respectively. This value is less than the current output by the solar cell at the battery’s open-circuit voltage for that state of charge, due to the internal resistance of the battery. As the battery is charged, its internal resistance can change,^39^ leading to a change in the slope of its I–V curve, thus changing the current which can be drawn. Once the battery is fully charged, the charge-transfer resistance of the electrode reaches its maximum value as no more lithium can be extracted from it. This leads to an increase in the battery resistance, resulting in a low current (*I*_3_) being drawn. A combination of these factors is what leads to a decrease in photocharging current with time.

In the fully charged state (time = *t*_3_ and voltage = *V*_3_), the potential of the battery is lower than the OCV of the solar cell. Thus, it continues to draw a current (*I*_3_) from the solar cell. As the battery is fully charged, this current will drive side reactions which are detrimental to the health of the battery. Since the current produced by a solar cell increases almost linearly with light intensity, this effect will be further exacerbated at higher light intensities, as described earlier. To minimize this, the shape of the battery charge–discharge curve and its upper cutoff voltage (*V*_3_) must be carefully considered, such that the battery operates in the low-current region of the solar cell I–V curve when *V*_3_ is reached. However, this may result in a trade-off with battery capacity if *V*_3_ is lower than the usual upper cutoff voltage.

It should be noted that this analysis is applicable specifically to systems where the catholyte is shared between the solar cell and the battery. In photobatteries with a solid cathode which is shared between the solar cell and the battery,^5,40^ the changing hole conductivity of the cathode as a function of SoC must also be considered alongside the change in battery resistance.

In summary, we establish relationships between the energy levels of photoelectrodes and anodes in photorechargeable batteries. To do this, we study the photocharging behavior of a dye-sensitized TiO_2_ photoelectrode when paired with two anodes which have intercalation potentials higher (LMO) and lower (LTO) than the E_Fc_ of TiO_2_. We show that LMO can be photocharged under illumination, but LTO cannot, as evidenced by a very small photocharging current and discharge capacity. Conversely, we vary the E_Fc_ of TiO_2_ by varying the light intensity and show that as long as E_Fc_ lies above the intercalation potential of the anode, photocharging can occur. Finally, we highlight the detrimental role of parasitic reactions in photoelectrochemical systems. We also provide some considerations for voltage–current matching in integrated photoelectrochemical systems. Although this study is carried out in a three-electrode architecture, we believe that the general principles are also applicable to two electrode photobatteries with bifunctional photoelectrodes. These results indicate a need to identify new mechanisms to understand the rise in OCV seen in zinc ion and lithium ion two-electrode photobatteries upon illumination.

## Data Availability

For the purpose of Open Access, the authors have applied a Creative Commons Attribution (CC BY) license to any Author Accepted Manuscript version arising from this submission. The data underlying this paper can be found at the University of Cambridge online repository [https://doi.org/10.17863/CAM.110645].

## Supporting Information Available

Materials and methods, additional photoelectrochemical measurements, and tables with data from literature (PDF)
