# Supplementary material for: What Makes a Photobattery Light-Rechargeable?
Source: ACS Energy Lett. 2024 Jul 23;9(8):4024–31. doi: 10.1021/acsenergylett.4c01350 (PMC11320653; doi:10.1021/acsenergylett.4c01350)
Supplement: Supplementary file 1 — nz4c01350_si_001.pdf [file nz4c01350_si_001.pdf]

Supplementary Material for "What Makes A Photobattery Light-Rechargeable?"

Arvind Pujari [1,2], Byung-Man Kim [2,*], Hooman Abbasi [2], Myeong-Hee Lee [3], Neil C. Greenham [1,*], and Michael De Volder [2,*]

[1]*Cavendish Laboratory, Department of Physics, University of Cambridge, Cambridge, CB3 0HE, United Kingdom.*

[2]*Institute for Manufacturing, Department of Engineering, University of Cambridge, Cambridge, CB3 0FE, United Kingdom.*

[3]*School of Energy and Chemical Engineering, Ulsan National Institute of Science & Technology, Ulsan, 44919, South Korea.*

*Address correspondence to kbm30003000@gmail.com (Byung-Man Kim), ncg11@cam.ac.uk (Neil C. Greenham) or mfld2@cam.ac.uk (Michael De Volder)

1. Materials and Methods

1.1 Preparation of TiO_2 Photoelectrode (PE)

FTO glass pieces with a thickness of 2.2 mm (Sigma Aldrich, surface resistivity ≈7 Ω/sq) and an area of 2.1cm x 1.7cm were successively sonicated in acetone (40 minutes), IPA (5 minutes) and deionized (DI) water (5 minutes). After drying, ultraviolet (UV) ozone treatment (BioForce Nanosciences) was conducted for 30 minutes to improve hydrophilicity of the surface. A compact TiO_2 blocking layer was deposited by placing the FTO glass in a 40 mM solution of $TiCl_4$ (Sigma Aldrich, 208566) in DI water for 35 minutes at 75 °C.

A mesoporous TiO_2 layer was deposited by screen-printing TiO_2 nanoparticles with an average size of 30 nm (30NR-D, Greatcell Solar Materials Pty. Ltd.) using a desktop screen printer (Wellcos, Korea). After drying at 120 °C for 5 minutes, a TiO_2 scattering layer (particle size > 100nm) was deposited by screen printing (Ti-Nanoxide, R/SP, Greatcell). After drying at 120 °C, the electrodes were annealed in air at 500 °C for 1 hour using a tube furnace (Carbolite Gero) to form crystalline TiO_2 layers. After cooling, the electrodes were immersed in 40mM $TiCl_4$ in DI H_2O at 75°C for 35 minutes followed by a second annealing at 500 °C for 1 hour. Two 0.5 mm holes were drilled (Dremel workstation) through the photoelectrode for electrolyte injection.

After cooling, the electrodes were immersed in a dye solution at room temperature for 18 h. The dye solution consisted of WS72, XY1b, and MS5 dyes (Dyenamo, Sweden). Each dye solution was made as follows; 0.1 mM WS72 and 1.0 mM chenodeoxycholic acid in toluene/EtOH (1:4 vol%), 0.1 mM XY1b and 1.0 mM chenodeoxycholic acid in t-BuOH/CH_3CN (1:1 vol%), and 0.1 mM MS5 and 1.0 mM chenodeoxycholic acid in t-BuOH/CH_3CN (1:1 vol%).

Finally, the solutions were mixed in a volumetric ratio of 3:1:1. The electrodes were then washed in EtOH to remove excess dye and dried with an air gun.

1.2 Preparation of $LiMn_2O_4$ (LMO)@Gn Storage Electrode

LMO@Gn was prepared using a prior procedure [1]. LMO@Gn electrode was composed of LMO@Gn as the active material, Super P (Timcal) carbon black as the conducting agent, and polyvinylidene fluoride (PVDF, Solef 5130) as the binder (weight ratio of 8:1:1), blade cast on an Al foil. Firstly, PVDF powder was completely dissolved in *N*-methyl pyrrolidinone (NMP). A ground mixture of LMO@Gn and Super P was finely dispersed into the PVDF/NMP solution with a mixer (ARE 310, THINKY Corp.). After casting the LMO@Gn slurry onto the Al foil using a doctor blade, it was vacuum dried at 110 °C for 10 hours, followed by natural cooling to room temperature. The loading density of LMO@Gn ranged from 3 to 3.8 mg cm^{-2}.

1.3 Preparation of $Li_4Ti_5O_{12}$ (LTO) Storage Electrode

The LTO electrode was prepared as follows. Initially, carbon nanotubes (CNT) were dispersed in deionized (DI) water using carboxymethyl cellulose (CMC) as a binder which was subsequently sonicated to achieve a stabilized gel-like slurry. Next, spherical LTO powder (BTR, China) was mixed with the CNT/CMC mixture using an ARM-310 Thinky mixer for 20 minutes. This LTO/CNT/CMC slurry was then coated onto a 17 µm thick aluminium foil and dried at 60 °C on a hot plate for 1 hour, followed by overnight drying in a vacuum oven at 60 °C. The resulting dried electrode had an average thickness of 40 µm and contained 2.9 mg cm^{-2} of active material.

1.4 Photobattery Assembly

Pt was deposited on LICGC™ through sputtering (Quorum Technologies Q150T) in a patterned fashion using a laser cut acrylic mask, as shown in Figure S2, to serve as the counter electrode of the solar cell. The Pt sputtered LICGC™ separator (discharge electrode, DE) was sealed against the photoelectrode using two precut 120 µm thermoplastic films (Surlyn™, Greatcell) by pressing on a hot plate at 120°C. The storage electrode (SE) was prepared by cutting strips of the prepare anode films (active area of 0.5cm x 0.3cm) and attaching them on a predrilled piece of glass using a thermoplastic film. The SE was then sealed against the other side of the DE using two precut 120 µm thermoplastic films. An electrolyte composed of 0.1 M LiI, 0.05M I_2, 0.05 M guanidine thiocyanate, 0.5 M of tert-butyl pyridine, and 0.6 M 1,2-dimethyl-3-propylimidazolium iodide (DMPII) in acetonitrile was injected between the PE and DE, and the holes were then sealed using a thermoplastic film and a cover glass. An electrolyte comprising of 0.1 M LiTFSI in ethylene carbonate (EC) and dimethyl carbonate (DMC) in a 1:1 ratio by volume was injected between the DE and SE with the holes sealed by the same procedure. Electronic contacts were soldered to the PE, SE and DE using an ultrasonic soldering machine (Sunbonder) and the entire device was sealed with potting epoxy (RS Components) and left to dry for 24 h.

1.5 Solar Cell Characterization

JV curves were measured by conducting cyclic voltammetry between the PE and DE with a scan rate of 50 mVs^{-1} for 1 sun measurements and 5 mV s^{-1} for indoor light measurements

using a Biologic VMP-3 potentiostat. For 1 sun measurements a solar simulator (Newport Oriel LSH-7320) was used as a light source with a 0.04 cm^2 black mask. For measurements with an LED, a white LED (Thorlabs) was used along with a 0.96 cm^2 black mask and a black box was used to block out background light.

1.6 Electrochemical Characterization

Photocharging measurements were carried out by conducting chronoamperometry (CA) at 0 V between the PE and SE using a Biologic VMP-3 potentiostat. All galvanostatic measurements were carried out by applying a current density of 50 µA cm^{-2} between the DE and LMO SE and a current density of 75 µA cm^{-2} between the DE and LTO SE.

The redox potential of I^-/I^{3-} and LMO were recorded by the average of redox peaks in cyclic voltammogram (marked by dotted line in Figure S2 and Figure S5). For I^-/I^{3-}, cyclic voltammetry was carried out at 100 mV s^{-1} scan rate in a 3-electrode system with Pt wires functioning as both working and counter electrodes, and Ag/Ag^+ as the reference electrode. The electrolyte was composed of 0.02M LiI, 0.01M I_2, and 0.1M LiTFSI in CH_3CN. For LMO, LMO cast on Al foil was used as the working electrode, and the electrolyte was 0.7M LiTFSI in acetonitrile. Ferrocene/ferrocenium pair (+0.63 V vs SHE) was used as an internal reference.

The highest occupied molecular orbital (HOMO) value of the WS72 dye was estimated from the half potential of cyclic voltammogram (Figure S4). All potential values were represented versus standard hydrogen electrode (SHE) by using an internal reference (Fc^+/Fc, +0.63 V vs SHE). For the cyclic voltammetry measurement, a dye-sensitized TiO_2 film on FTO glass, Pt wire, and Ag/Ag^+ were used as working, counter, reference electrodes, respectively. The electrolyte was prepared by dissolving 0.1M of LiTFSI in acetonitrile. To fabricate the working electrode, TiO_2 solution (30NR-D:EtOH:Triton X-100 / 3:6:1) was spin-coated on FTO glass at 1500 rpm for 40 s, followed by annealing at 500 °C for 30 min in air. After cooling down to 80 °C, the electrode was immersed in a 0.0001 M WS72 dye solution (toluene:EtOH, 1:4 vol%) with 0.001 M chenodeoxycholic acid (CDCA) as the co-adsorbate at room temperature for 17h.

1.7 Optical Characterization

The dye absorption was measured by using dye-sensitized TiO_2 films and the UV-vis spectra were recorded using a Perkin Elmer Lambda-750 UV-Vis-NIR Spectrometer.

2. Supplementary Table

Table S1. A summary of the conduction band potential of the photocathode and anode plating potentials of some two-electrode photobatteries reported in literature.

Photocathode	E_{CB} (vs SHE)	Anode	Anode plating potential (vs SHE)	Ref	Comments
Dye-sensitized $LiFePO_4$	-1.44 V	Li	- 3 V	[2]	Trace amounts of oxygen acted as electron acceptors.
Lead-halide perovskite	- 1.44 V	Li	-3 V	[3]	

Lead-free perovskite	- 1 V	Li	-3 V	[4]	
V_2O_5/P3HT	- 2 V	Li	- 3 V	[5]	
V_2O_5/Carbon Cloth	- 2 V	Li	- 3 V	[6]	
V_2O_5/P3HT	- 2 V	Zn	- 0.76 V	[7]	Photocharging theoretically possible.
MoS_2	- 0.2 V	Zn	- 0.76 V	[8]	
VO_2	- 0.6 V	Zn	- 0.76 V	[9]	
VO_2-ZnO	- 0.6 V	Zn	- 0.76 V	[10]	
TiS_2-TiO_2	+ 0.2 V	Li	- 3 V	[11]	
MoS_2-MoO_3	- 0.2 V	Li	- 3 V	[12]	
LiV_2O_5	- 0.1 V	Li	- 3 V	[13]	
TiO_2@MoS_2	- 2.2 V	Na	- 2.76 V	[14]	
Fe_2O_3	- 1 V	Li	- 3 V	[15]	
TiO_2-Cu_2O	- 0.4 V	Li	- 3 V	[16]	
PbPA-$CsPbBr_3$	+ 0.2 V	Li	- 3 V	[17]	Trace amounts of oxygen acted as electron acceptors.
$ZrSe_2$	~- 0.2 V	Cu	0.34 V	[18]	Photocharging theoretically possible and photocharging current recorded.
PANI	- 1.4 V	Al	-1.66 V	[19]	
CuTTP dye - CuV_2O_5 – TiO_2	- 0.2 V	Zn	-0.76 V	[20]	Photoelectrons are injected into TiO_2 - Fermi level too low.
$MoSe_2$	- 0.3 V	Zn	-0.76 V	[21]	
V_2O_3	- 0.5 V	Zn	-0.76 V	[22]	

3. Supplementary Figures

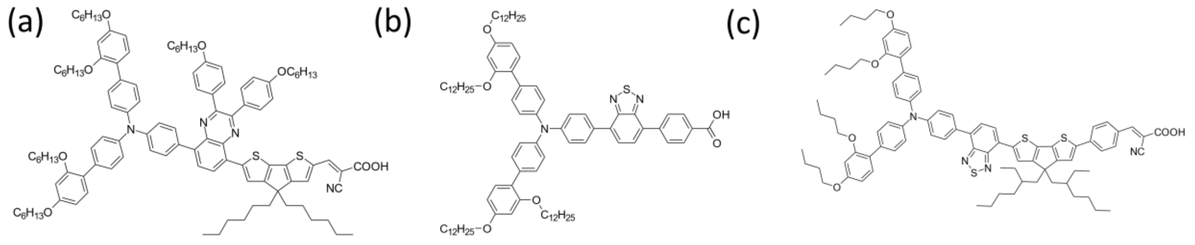

WS72 MS5 XY1b

Figure S1. The structure of the three dyes used for the co-sensitization of TiO_2 photoelectrode (a) WS72, (b) MS5 and (c) XY1b.

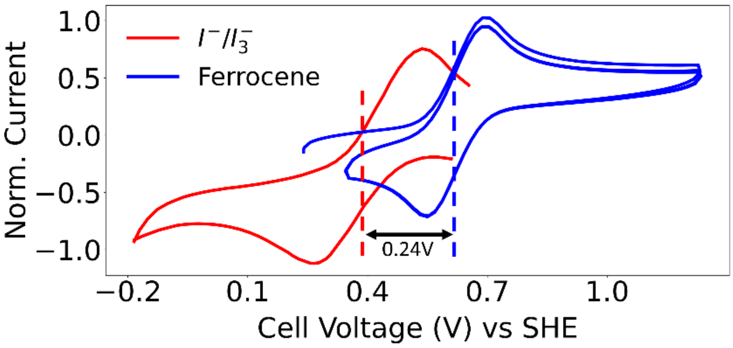

Figure S2. CV curves of the triiodide redox couple and ferrocene redox couple in acetonitrile to obtain its redox potential. The redox potential of ferrocene (Fc^+/Fc, +0.63 V vs SHE) was used to obtain the redox potential of the triiodide redox mediator vs SHE.

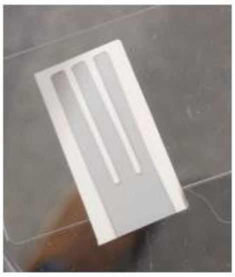

Patterned Pt
sputtered on LISICON™

Figure S3. An image of the patterned Pt sputtered on LISICON™.

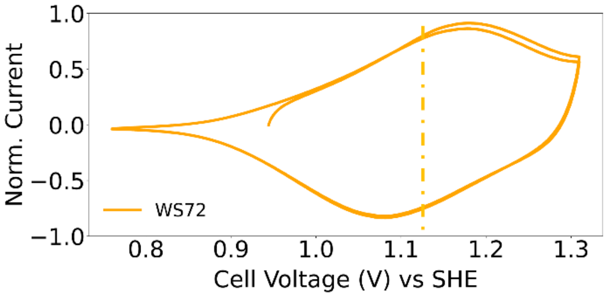

Figure S4. CV curves of the WS72 dye in a solution of LiTFSI in acetonitrile. The half potential (at +1.13 V vs SHE) represents the HOMO level of the dye. All potential values are represented versus standard hydrogen electrode (SHE) by using internal reference (Fc^+/Fc, +0.63 V vs SHE).

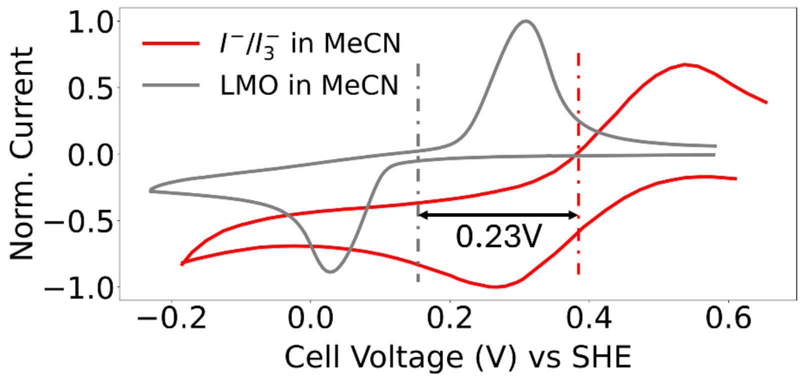

Figure S5. CV curves of the LMO anode and I_3^-/I^- redox couple in a solution of LiI in acetonitrile. The distance between the half potentials is about + 0.23 V, which is in good agreement with the discharge potential we obtained, indicating that the photocharging reaction is Li^+ intercalation into LMO.

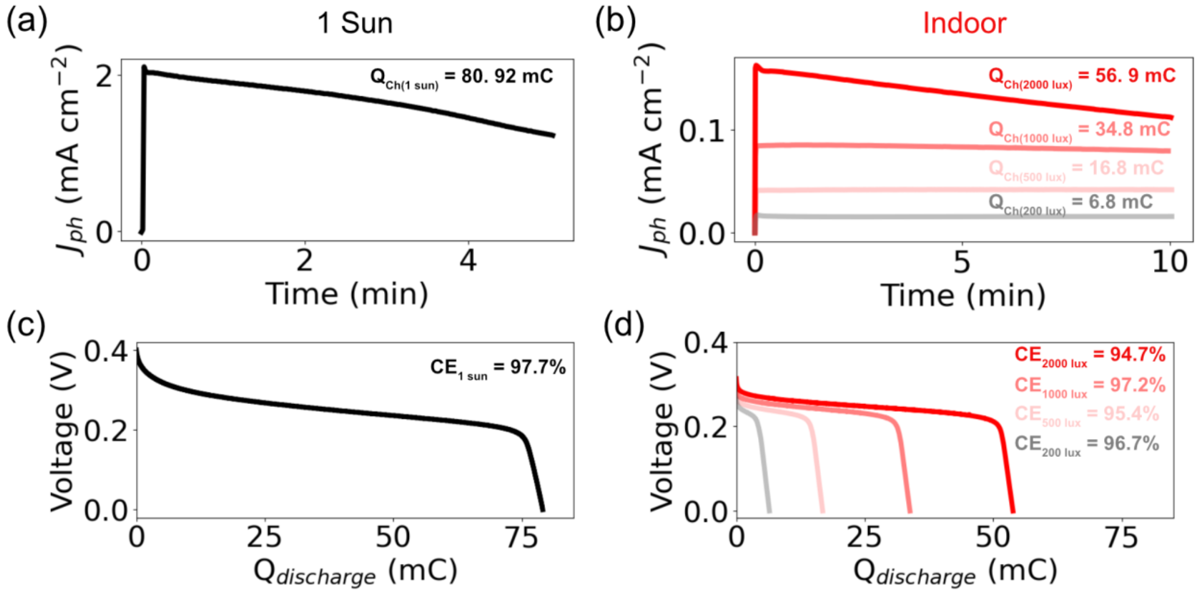

Figure S6. Five consecutive photocharging-discharging cycles at different light intensities. (a) Photocharging of LMO under 1 sun illumination for 5 minutes. (b) Photocharging of LMO under 2000 lux, 1000 lux, 500 lux and 200 lux for 10 minutes. (c) Discharging after photocharging in (a) at 14 µA cm^{-2}. (d) Discharging after photocharging in (b) at 4 µA cm^{-2}. In both cases, a coulombic efficiency between 94-97% is seen for all cycles.

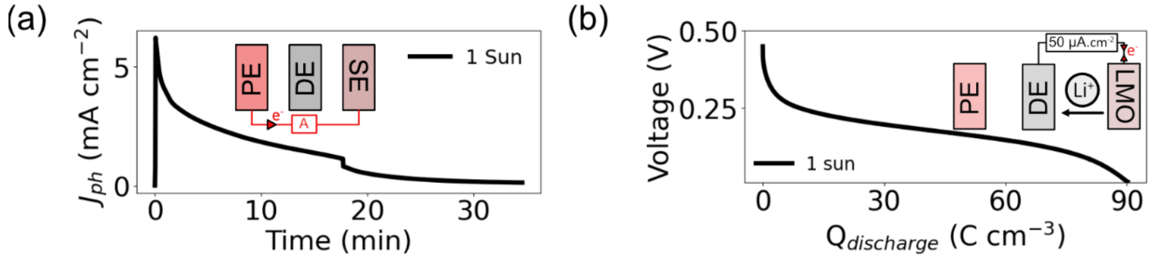

Figure S7. (a) Long term photocharging of the I_3^-/I^- vs LMO battery under 1 sun conditions, with a sharp decrease in current seen during the photocharging process. (b) Discharge curves after the long-term photocharging, with the same discharge capacity as that under 200 lux and 1000 lux long-term photocharging obtained.

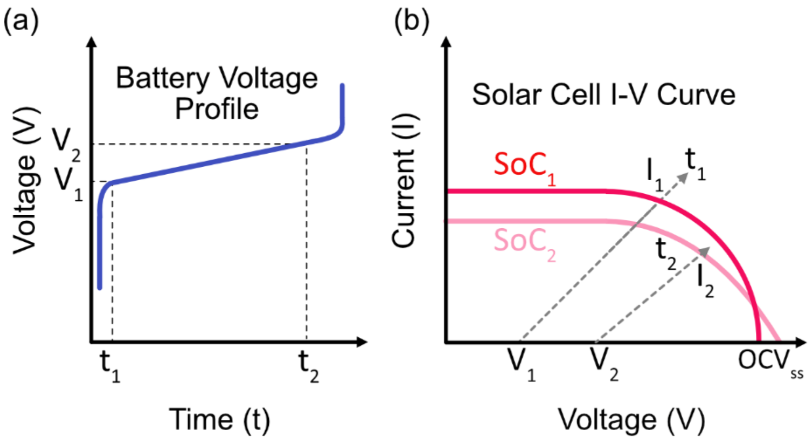

Figure S8. (a) Sketch of a battery voltage vs time curve with a sloping voltage profile. (b) I-V curves of the solar cell at two different SoCs, with the battery I-V curves superimposed. As the battery charges, the lithium-ion content in the solar cell electrolyte decreases, causing an increase in OCV_{ss} but a decrease in current [23]. This sketch illustrates that the changing I-V curve of the solar cell as a function of SoC is also a contributing factor to the decrease in photocurrent and must be considered for I-V matching in photobatteries. It should be noted that this figure is a sketch for illustrative purposes and does not contain real data.

4. References

[1] M.-H. Lee *et al.*, 'Electrochemically Induced Crystallite Alignment of Lithium Manganese Oxide to Improve Lithium Insertion Kinetics for Dye-Sensitized Photorechargeable Batteries', *ACS Energy Lett.*, vol. 6, no. 4, pp. 1198–1204, Apr. 2021, doi: 10.1021/acsenergylett.0c02473.

[2] A. Paolella *et al.*, 'Light-assisted delithiation of lithium iron phosphate nanocrystals towards photo-rechargeable lithium ion batteries', *Nat. Commun.*, vol. 8, no. 1, p. 14643, Apr. 2017, doi: 10.1038/ncomms14643.

[3] S. Ahmad, C. George, D. J. Beesley, J. J. Baumberg, and M. De Volder, 'Photo-Rechargeable Organo-Halide Perovskite Batteries', *Nano Lett.*, vol. 18, no. 3, pp. 1856–1862, Mar. 2018, doi: 10.1021/acs.nanolett.7b05153.

[4] N. Tewari, S. B. Shivarudraiah, and J. E. Halpert, 'Photorechargeable Lead-Free Perovskite Lithium-Ion Batteries Using Hexagonal $Cs_3Bi_2I_9$ Nanosheets', *Nano Lett.*, vol. 21, no. 13, pp. 5578–5585, Jul. 2021, doi: 10.1021/acs.nanolett.1c01000.

[5] B. D. Boruah, B. Wen, and M. De Volder, 'Light Rechargeable Lithium-Ion Batteries Using V_2O_5 Cathodes', *Nano Lett.*, vol. 21, no. 8, pp. 3527–3532, Apr. 2021, doi: 10.1021/acs.nanolett.1c00298.

[6] M. Wilhelm *et al.*, 'Carbon-Coated Electrospun V_2O_5 Nanofibers as Photoresponsive Cathode for Lithium-Ion Batteries', *Adv. Eng. Mater.*, vol. 25, no. 1, p. 2200765, 2023, doi: 10.1002/adem.202200765.

[7] B. Deka Boruah, A. Mathieson, B. Wen, S. Feldmann, W. M. Dose, and M. D. Volder, 'Photo-rechargeable zinc-ion batteries', *Energy Environ. Sci.*, vol. 13, no. 8, pp. 2414–2421, 2020, doi: 10.1039/D0EE01392G.
[8] B. D. Boruah, B. Wen, and M. De Volder, 'Molybdenum Disulfide–Zinc Oxide Photocathodes for Photo-Rechargeable Zinc-Ion Batteries', *ACS Nano*, vol. 15, no. 10, pp. 16616–16624, Oct. 2021, doi: 10.1021/acsnano.1c06372.
[9] B. D. Boruah *et al.*, 'Vanadium Dioxide Cathodes for High-Rate Photo-Rechargeable Zinc-Ion Batteries', *Adv. Energy Mater.*, vol. 11, no. 13, p. 2100115, 2021, doi: 10.1002/aenm.202100115.
[10] B. D. Boruah and M. D. Volder, 'Vanadium dioxide–zinc oxide stacked photocathodes for photo-rechargeable zinc-ion batteries', *J. Mater. Chem. A*, vol. 9, no. 40, pp. 23199–23205, 2021, doi: 10.1039/D1TA07572A.
[11] A. Kumar *et al.*, 'Photo-Rechargeable Li-Ion Batteries using TiS_2 Cathode', *Small*, vol. 19, no. 38, p. 2303319, 2023, doi: 10.1002/smll.202303319.
[12] A. Kumar, P. Thakur, R. Sharma, A. B. Puthirath, P. M. Ajayan, and T. N. Narayanan, 'Photo Rechargeable Li-Ion Batteries Using Nanorod Heterostructure Electrodes', *Small*, vol. 17, no. 51, p. 2105029, 2021, doi: 10.1002/smll.202105029.
[13] J. Wang, Y. Wang, C. Zhu, and B. Liu, 'Photoinduced Rechargeable Lithium-Ion Battery', *ACS Appl. Mater. Interfaces*, vol. 14, no. 3, pp. 4071–4078, Jan. 2022, doi: 10.1021/acsami.1c20359.
[14] J. Li *et al.*, 'Dual-Functional Z-Scheme TiO_2@MoS_2@NC Multi-Heterostructures for Photo-Driving Ultrafast Sodium Ion Storage', *Angew. Chem. Int. Ed.*, vol. 62, no. 34, p. e202303056, 2023, doi: 10.1002/anie.202303056.
[15] S. Chamola and S. Ahmad, 'High Performance Photorechargeable Li-Ion Batteries Based on Nanoporous Fe_2O_3 Photocathodes', *Adv. Sustain. Syst.*, vol. 7, no. 6, p. 2300043, 2023, doi: 10.1002/adsu.202300043.
[16] I. Ciria-Ramos, E. J. Juarez-Perez, and M. Haro, 'Solar Energy Storage Using a Cu_2O-TiO_2 Photocathode in a Lithium Battery', *Small*, vol. 19, no. 28, p. 2301244, 2023, doi: 10.1002/smll.202301244.
[17] J. Pan *et al.*, 'Efficient Bifunctional Photoelectric Integrated Cathode for Solar Energy Conversion and Storage', *ACS Nano*, vol. 17, no. 21, pp. 21360–21368, Nov. 2023, doi: 10.1021/acsnano.3c06096.
[18] H. Tributsch, 'Photo-intercalation: Possible application in solar energy devices', *Appl. Phys.*, vol. 23, no. 1, pp. 61–71, Sep. 1980, doi: 10.1007/BF00899572.
[19] L.-L. Chen, X. Bu, W.-L. Song, H.-S. Chen, W. Wang, and S. Jiao, 'Stable Photo-Rechargeable Al Battery for Enhancing Energy Utilization', *Adv. Mater.*, p. 2306701, doi: 10.1002/adma.202306701.
[20] S. Naskar *et al.*, 'A Förster resonance energy transfer enabled photo-rechargeable battery with an energetically misaligned Cu-porphyrin dye/Cu:V_2O_5 photocathode', *J. Mater. Chem. A*, vol. 12, no. 25, pp. 15203–15226, 2024, doi: 10.1039/D3TA06248A.
[21] X. Chen, A. Zhang, H. Zou, L. Li, Q. Zhu, and W. Zhang, 'Defect engineering modulated $MoSe_2$ cathode achieves highly effective photo-responsive zinc ion battery', *Energy Storage Mater.*, vol. 70, p. 103457, Jun. 2024, doi: 10.1016/j.ensm.2024.103457.
[22] Y. Zhao *et al.*, 'Carbon superstructure-supported half-metallic V_2O_3 nanospheres for high-efficiency photorechargeable zinc ion batteries', *Angew. Chem. Int. Ed.*, p. e202408218, doi: 10.1002/anie.202408218.
[23] H.-G. Han *et al.*, 'Influence of the Lithium-Ion Concentration in Electrolytes on the Performance of Dye-Sensitized Photorechargeable Batteries', *ACS Appl. Mater. Interfaces*, vol. 15, no. 34, pp. 40378–40384, Aug. 2023, doi: 10.1021/acsami.3c05250.
